# *SlMYB12* Regulates Flavonol Synthesis in Three Different Cherry Tomato Varieties

**DOI:** 10.1038/s41598-018-19214-3

**Published:** 2018-01-25

**Authors:** Shaoli Wang, Zhaohui Chu, Ru Jia, Fei Dan, Xiangling Shen, Yang Li, Xinhua Ding

**Affiliations:** 1 State Key Laboratory of Crop Biology, Shandong Provincial Key Laboratory for Biology of Vegetable Diseases and Insect Pests, Shandong Agricultural University, Taian, 271018 Shandong P. R. China; 2 Anhui Biothun Biotechnology Company, Hefei, Anhui 230088 P. R. China; 3 Biotechnology Research Center, China Three Gorges University, Yichang City, Hubei 443002 P. R. China

## Abstract

Cherry tomato (*Lycopersicon esculentum* M.) is considered a healthy fruit worldwide due to its wide range of nutrients. Flavonol, one of the major nutrients in cherry tomato, has antioxidant and cell-modulating properties. In this study, we showed a correlation between the expression of *SlMYB12* and flavonol content (R^2^ = 0.922). To characterize the function of *SlMYB12*, *SlMYB12*-overexpressing transgenic tomato plants were generated in three different cherry tomato varieties. Significant increases in flavonol content and flavonol biosynthetic gene expression were identified in *SlMYB12*-overexpressing plants. Therefore, we suggest that *SlMYB12* plays a positive role in the flavonol biosynthesis pathway in cherry tomatoes, which further indicates a potential role as a marker in analyzing flavonol content in different cherry tomato varieties.

## Introduction

Currently, mounting scientific evidence supports the fact that fruits and vegetables can reduce the incidence and mortality of chronic disease, which has risen considerably in the 21st century^1–4^. Cherry tomato (*Lycopersicon esculentum* M.) is considered part of a healthy diet due to its wide range of nutrients^5^. Flavonol, one of the richest phytochemicals in cherry tomato, is highly associated with human health due to its antioxidant and cell-modulating properties. Flavonols can be divided into several types, such as quercetin rutinoside (rutin), kaempferolrutinoside, and naringenin chalcone (NC)^6,7^. Rutin, which scavenges free radicals in organisms, has potential anti-inflammatory, anticarcinogenic, and antimicrobial effects by suppressing cellular immunity^8–11^. A previous study has demonstrated that rutin also has an effect on regulating the brain cholinergic system when co-administered with priacetam and phenytoin^12^. Apart from these, rutin shows an ability to control brain neurotransmitters and protect against heart disease^13,14^. Kaempferolrutinoside can increase free radical scavenging capacity by regulating the expression of antioxidant enzymes and transcription factors^15^. It also has a positive effect on controlling the migration of human keratinocyte cells through the FAK/Akt pathway, so it can be used as an active agent for wound healing^16–18^. Naringenin chalcone, the major active component in tomato skin^19^, has anti-allergic activity via inhibiting histamine release *in vivo*, so it protects human cells against the hepatitis C virus and relieves perennial allergic rhinitis^20–23^.

**Figure 1:**
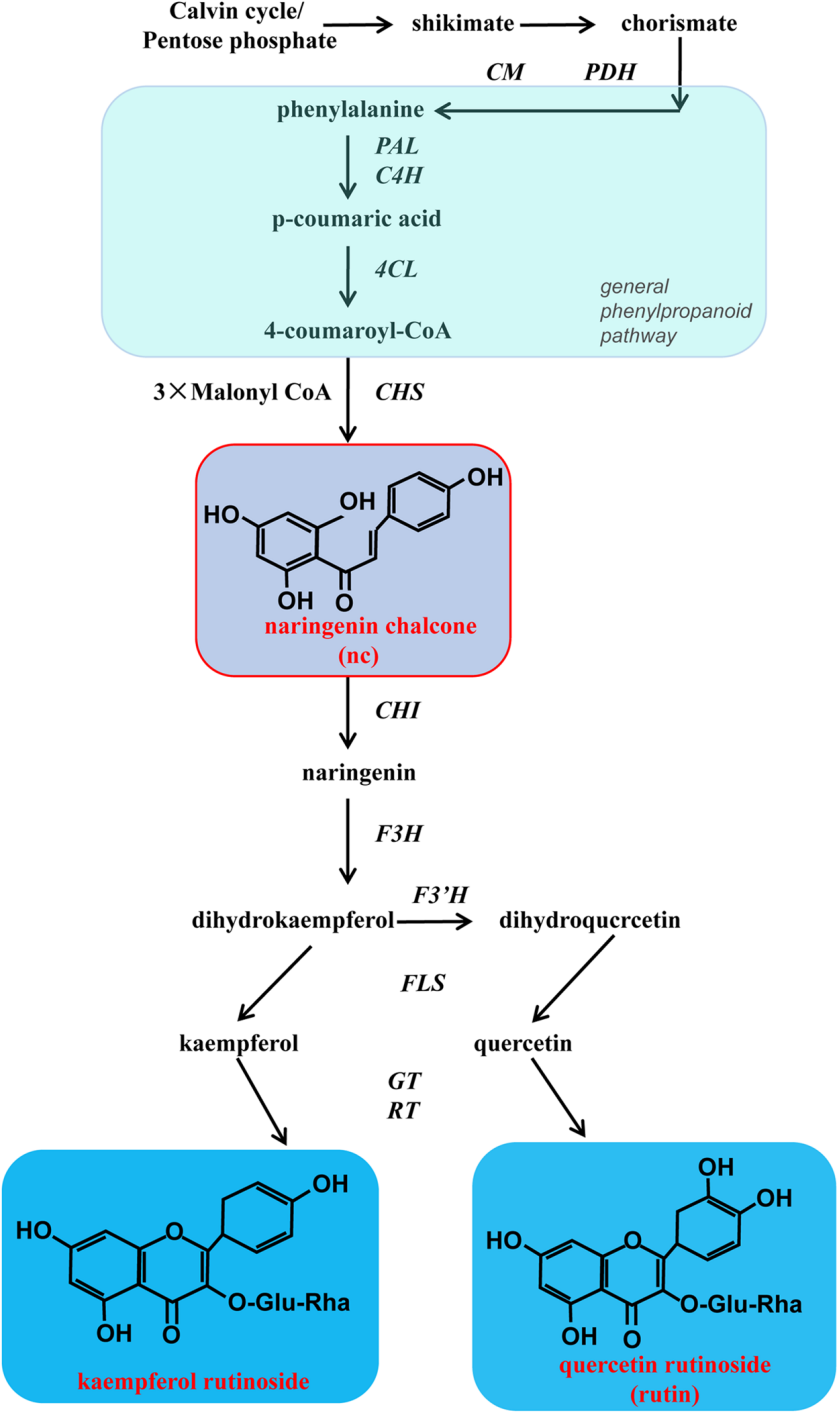
Flavonol biosynthesis pathway in tomato. The enzymes involved in each step are abbreviated: CM, chorismate mutase; PDH, prephenate aminotransferase; PAL, phenylalanine ammonialyase; C4H, cinnamate 4-hydroxylase; 4CL, 4-hydroxycinnamoyl CoA ligase; CHS, chalcone synthase; CHI, chalcone isomerase; F3H, flavanone-3-hydroxylase; F3′H, flavonoid-3′-hydrosylase; FLS, flavonol synthase; GT, flavonol-3-glucosyltransferase; RT, flavonol-3-glucoside-rhamnosyltransferase.

The MYB family is a large family of transcriptional regulators in plants. It has been proved that MYB proteins can act as key components in the regulation of specific genes^24^. Among these MYB proteins, some members play an important role in flavonoid biosynthesis by regulating the expression of enzymes in the biosynthetic pathway (Fig. 1)^25,26^. For example, *Arabidopsis thaliana* transcription factor *AtMYB12*, a flavonol-specific activator of flavonol biosynthesis, positively regulates the expression of flavonol biosynthetic genes^27–30^. In *AtMYB12*-expressing ripe tomato fruit, total flavonols were up to 70-fold higher than those of controls, and the total hydrophilic antioxidant capacity of fruit was enhanced up to five fold^7^. *AtMYB11*, a homolog of *AtMYB12*, regulated the content of flavonol in tomato and tobacco. Constitutive expression of *AtMYB11* enhanced the expression of key genes in the phenylpropanoid biosynthesis pathway^31^. *AtMYB111*, another homolog of *AtMYB12*, positively regulated the biosynthesis of flavonol in tobacco^32^. Tomato gene *SlMYB12* showed 80% amino acid identity with *AtMYB12*^7^, and down-regulation of *SlMYB12* led to pink coloration in tomato fruit, this effect is due to the absence of naringenin chalcone that leads to yellow coloration^33^. In wild-type Micro-Tom fruit, *SlMYB12* showed the highest transcript level at the ripening stage, which is highly correlated with the most rapid increase of flavonol. Moreover, *SlMYB12* showed obviously higher expression in fruit skins, associated with the higher accumulation of flavonol, while *SlMYB12* showed lower expression in fresh fruit with lower accumulation^7^. These results suggested probable correlations between *SlMYB12* and flavonol biosynthesis in tomato. However, the detailed function of the tomato endogenous gene *SlMYB12* in flavonol biosynthesis regulation is still unknown.

In previous studies, flavonol contents have been characterized in different tomato varieties^34–37^; however, no study has previously reported on the correlation between the original expression level of *SlMYB12* and the behavior of flavonols in varieties of cherry tomatoes. Furthermore, this study presents a potential role of *SlMYB12* as a marker to monitor the flavonol content in varieties of cherry tomatoes. Moreover, we also generated *SlMYB12*-overexpressing transgenic lines in three different cherry tomato varieties driven by the E8 fruit-specific promoter, and the further investigation of these overexpression lines exhibited the potential regulator or target genes that regulated by *SlMYB12*. The analysis of antioxidant capacity was performed not only among varieties of cherry tomato cultivars but also in three *SlMYB12* overexpression lines, and these results further confirmed the potential function of *SlMYB12* as a monitoring marker for flavonol content and antioxidant capacity in tomato fruits.

## Results

### Flavonol contents in three cherry tomato cultivars

**Figure 2:**
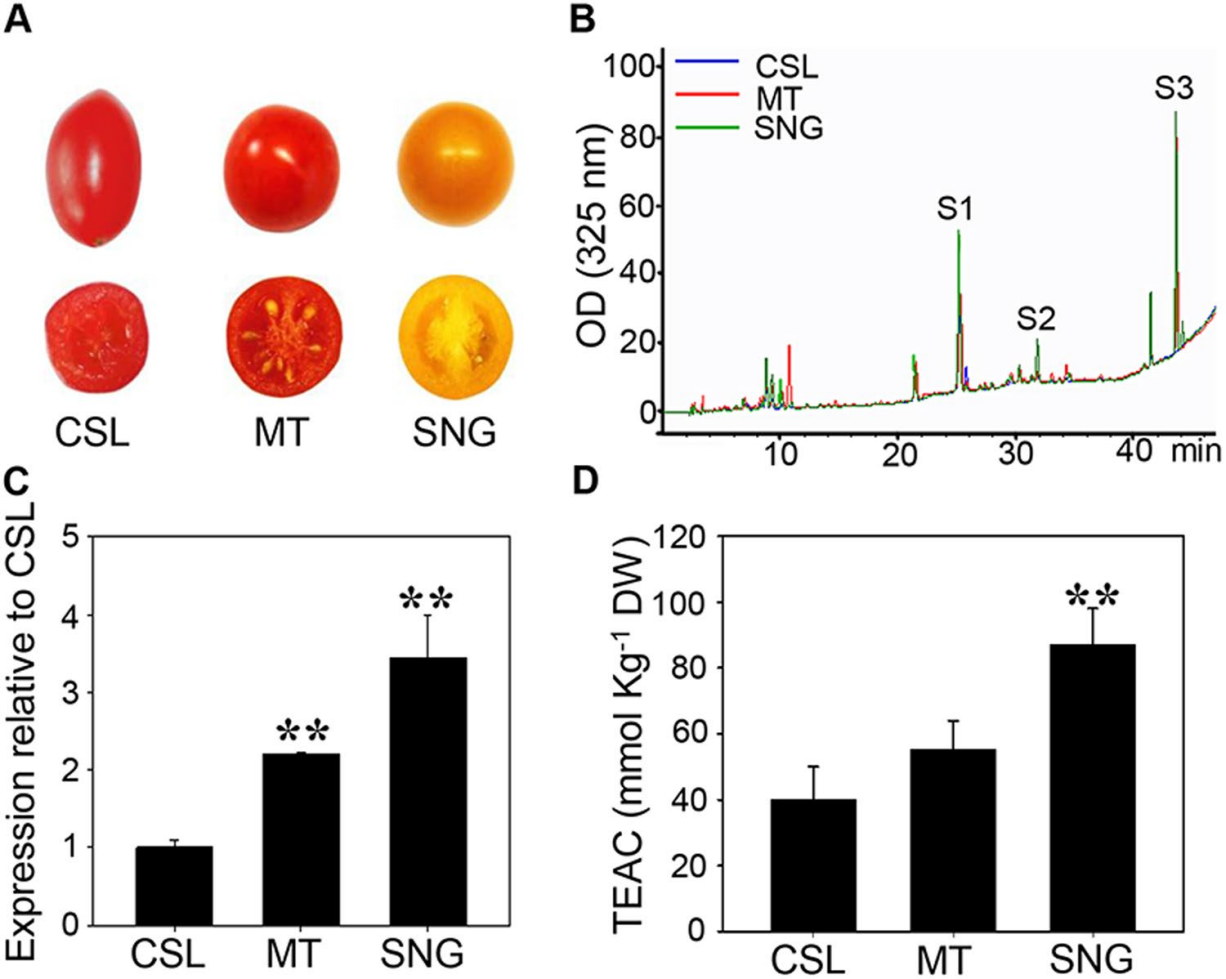
Phenotypes of three cherry tomato cultivars. (**A**) Phenotype of fruit in three cherry tomato cultivars. CSL, Csl09-03; MT, Micro-Tom; SNG, Sheng Nv-Guo. (**B**) HPLC analysis of extracts from three cherry tomato cultivars. S1, quercetin rutinoside (rutin); S2, kaempferol rutinoside; S3, naringenin chalcone. (**C**) Relative expression levels of *SlMYB12* in three cherry tomato cultivars. Each value was normalized to *ASR1* expression and is indicated as the mean ± standard deviation of three experimental replicates. (**D**) Total antioxidant capacity in three cherry tomato cultivars. Fresh and skin antioxidant activities in mature tomato fruits of each cultivar. Three different tomato fruits of each cultivar were pooled for detection. Each value represents repeated four times independent experiments, and the vertical bars expressed the arithmetic means ± standard deviations (SD). **P < 0.01.

There are different phenotypes among the three tomato cultivars: CSl09–03, Micro-Tom and Sheng Nv-Guo. The fruit of CSl09–03 is long-elliptical with a deep pink color, Micro-Tom is spherical with a bright red color, and Sheng Nv-Guo is spherical with a bright orange color (Fig. 2A). Fruits of each cultivar were harvested and characterized by HPLC on a dry-weight basis for their major flavonol contents (quercetin rutinoside (rutin), kaempferolrutinoside and naringenin chalcone). Based on these analyses, all three kinds of flavonol in fruit skins were quantified with purified standards. By standardizing to fruit skin dry weight, we found that tomato variety Csl09–03 contains the lowest flavonol contents, including rutin (110.37 µg/g), kaempferolrutinoside (30.15 µg/g) and naringenin chalcone (276.14 µg/g), respectively. In contrast, the total flavonol content in Micro-Tom (1116.12 µg/g) is approximately 2.68-fold higher than that in Csl09–03 (416.66 µg/g). The highest flavonol content was observed in variety Sheng Nv-Guo, which contains 1350.20 µg/g rutin, 330.69 µg/g kaempferolrutinoside and 1687.59 µg/g naringenin chalcone. Overall, an average of 3368.48 µg flavonol was detected in 1 g DW in Sheng Nv-Guo, which was 8.08-fold higher than that in CSl09-03 (Table 1, Fig. 2B). All these results indicated that phenotypes and flavonol levels are diverse in different wild-type tomato varieties.

**Table 1:** Quantification of major flavonols in three wild-type cherry tomato variety peels.

	QueRut^d^ (μg g^−1^ DW^g^)	KaeRut^e^ (μg g^−1^ DW)	NC^f^ (μg g^−1^ DW)
CSL^a^	110.37 ± 0.25	30.15 ± 0.01	276.14 ± 5.89
MT^b^	450.46 ± 0.17	230.40 ± 0.10	435.26 ± 3.23
SNG^c^	1350.20 ± 1.50	330.69 ± 0.91	1687.59 ± 10.23

^a^CSL, wild-type (Csl09-03) tomato peels; ^b^MT, wild-type (Micro Tom) tomato peels; ^c^SNG, wild-type (Sheng Nv-Guo) tomato peels; ^d^QueRut, quercetin rutinoside (rutin); ^e^KaeRut, kaempferolrutinoside; ^f^NC, naringenin chalcone; ^g^DW, dry weight.

### Expression of *SlMYB12* is highly correlated with flavonol biosynthesis in three tomato varieties

To investigate the correlation between the expression of *SlMYB12* and flavonol content, we used quantitative RT-PCR to analyze the expression level of *SlMYB12* in different tomato varieties. The expression levels of *SlMYB12* in Micro-Tom and Sheng Nv-Guo were 2.2-fold and 3.4-fold higher than that in Csl09-03 (Fig. 2C). Interestingly, these results were consistent with the flavonol contents in each tomato variety, which may indicate a correlation between the transcript levels of *SlMYB12* and the flavonol contents. Further analysis indicated that a strong correlation was found between the transcriptional levels of *SlMYB12* and the flavonol contents in three tomato varieties. All these results demonstrated that flavonol contents were highly associated with the expression level of *SlMYB12* in different tomato varieties.

**Figure 3:**
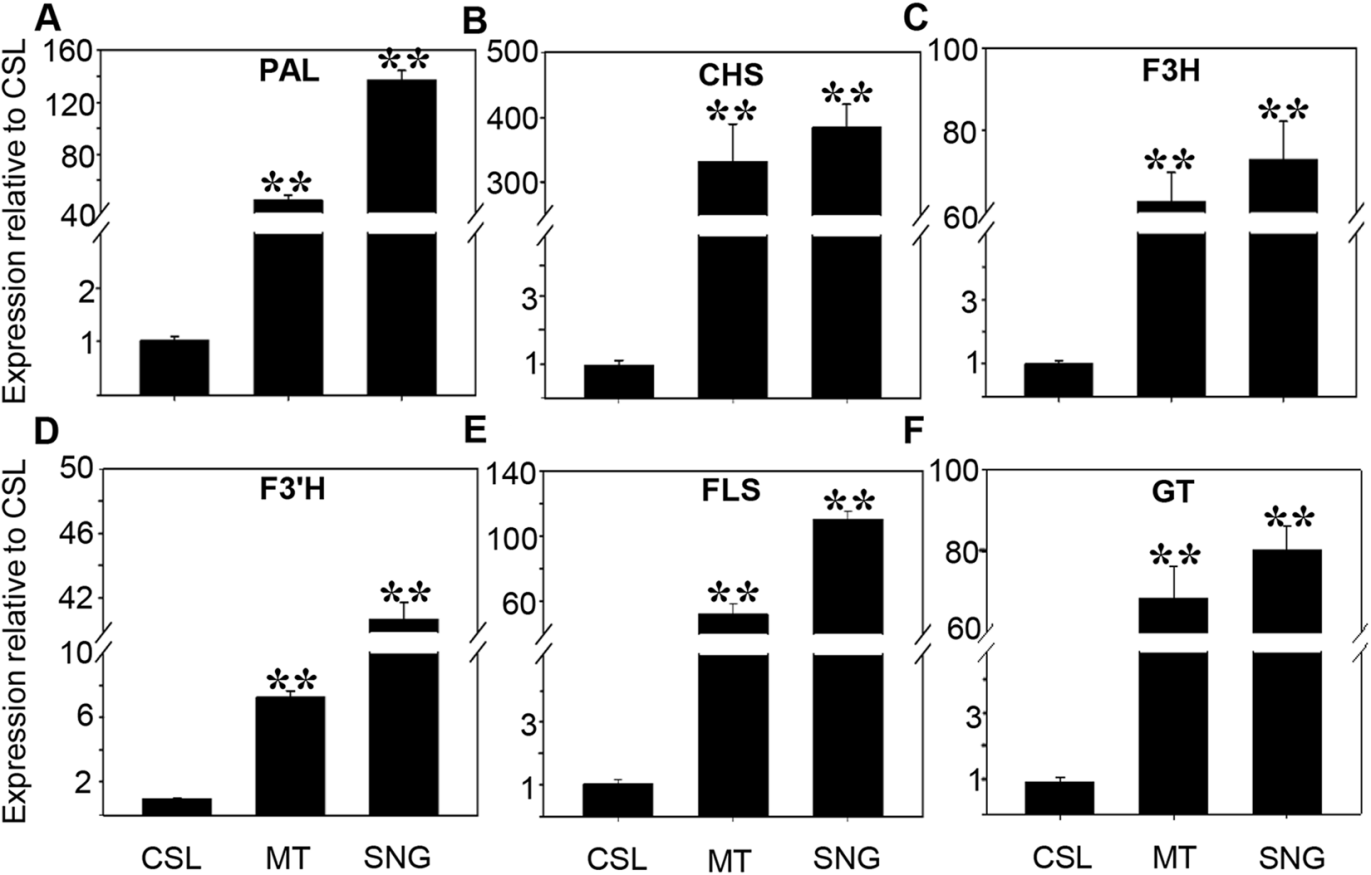
Analysis of transcript levels of the main genes in flavonol metabolism in three cherry tomato cultivars by qRT-PCR. Analyzed genes are described in Fig. 1. (**A**) PAL, phenylalanine ammonialyase; (**B**) CHS, chalcone synthase; (**C**) F3H, flavanone-3-hydroxylase; (**D**) F3′H, flavonoid-3′-hydroxylase; (**E**) FLS, flavonol synthase; (**F**) GT, flavonol-3-glucosyltransferase. Two or three fruits were pooled from each plant. Each value was normalized to the gene expression in cultivar CSL and is indicated as the mean ± standard deviation of three experimental replicates. **P < 0.01.

To explore the potential role of *SlMYB12* in the flavonol biosynthesis pathway, quantitative RT-PCR was performed to detect the expression of flavonol biosynthesis genes in three tomato cultivars. Generally, the expression of PAL (phenylalanine ammoniumlyase), a proposed catalytic enzyme in the phenylpropanoid metabolic pathway, was 44.49-fold higher in Micro-Tom and 137.17-fold higher in Sheng Nv-Guo than that in Csl09-03 (Fig. 3A); CHS (chalcone synthase), which regulates the biosynthesis of naringenin chalcone, was 331.57-fold and 384.43-fold higher in Micro-Tom and Sheng Nv-Guo than that in Csl09-03 (Fig. 3B). Similarly, other flavonol biosynthesis genes, including F3H (flavanone-3-hydroxylase), F3′H (flavonoid-3′-hydroxylase), FLS (flavonol synthase) and GT (glucosyl transferase), showed variably increased expression levels compared with the expression level in CSl09-03. For example, the expression levels of these 4 enzymes were 62.72-, 7.20-, 51.93- and 68.34-fold higher in Micro-Tom, and 72.80-, 40.80-, 110.48- and 78.49-fold higher in Sheng Nv-Guo (Fig. 3C–F).

Flavonol is thought to be highly associated with human health due to its antioxidant capacity, and the antioxidant capacity in these three tomato varieties were measured by TEAC (Trolox equivalent antioxidant capacity) assay. The antioxidant capacity of the flavonols in Micro-Tom was 1.38-fold higher than that in CSl09-03, and the antioxidant capacity in Sheng Nv-Guo was 2.18-fold higher (Fig. 2D). These results were also highly consistent with the expression level of *SlMYB12* and the flavonol contents in the three tomato varieties (R^2^ = 0.922). As correlation analysis R^2^ = 0.922 of *SlMYB12* expression level and flavonols content is examined in the only three wild-type tomato varieties, the number of analyzed cultivars is small to evaluate, we used 10 more wild-type cultivars to examine the correlation. The skins of each cultivar were characterized by HPLC on a fresh-weight basis to analyze their major flavonol contents. Cultivar 1 had the lowest flavonol content (469.64 µg g^−1^), while the highest content was observed in cultivar 10 (15037.83 µg g^−1^) (Table S2). Quantitative RT-PCR was used to analyze the expression level of *SlMYB12*, and the expression levels in cultivars 2 to 10 were higher than that in cultivar 1. Based on this result, the expression level of *SlMYB12* in cultivar 1 was normalized to 1, and the expression levels in cultivars 2 to 10 were expressed as fold changes (Table S2). A correlation analysis of *SlMYB12* expression levels and flavonol contents among the ten tomato cultivars were conducted, and the correlation coefficient was also high, R^2^ = 0.929 (Fig. S1). These results demonstrated that *SlMYB12* expression level was highly correlated with flavonol content in tomato fruits.

Overall, high expression of *SlMYB12* in a tomato variety elevated its antioxidant capacity, which depended on higher flavonol content. Correlation analyses of flavonol content and tomato fruit antioxidant capacity among tomato varieties were conducted, and the correlation coefficients were very high. These results demonstrated that antioxidant capacity was positively associated with flavonol content in tomato varieties. Furthermore, the expression of *SlMYB12* was highly correlated with altered expression levels of the major genes in the flavonol synthesis pathway.

### Overexpression of SlMYB12 leads to different phenotypes and enhanced flavonol contents in three tomato cultivars

**Figure 4:**
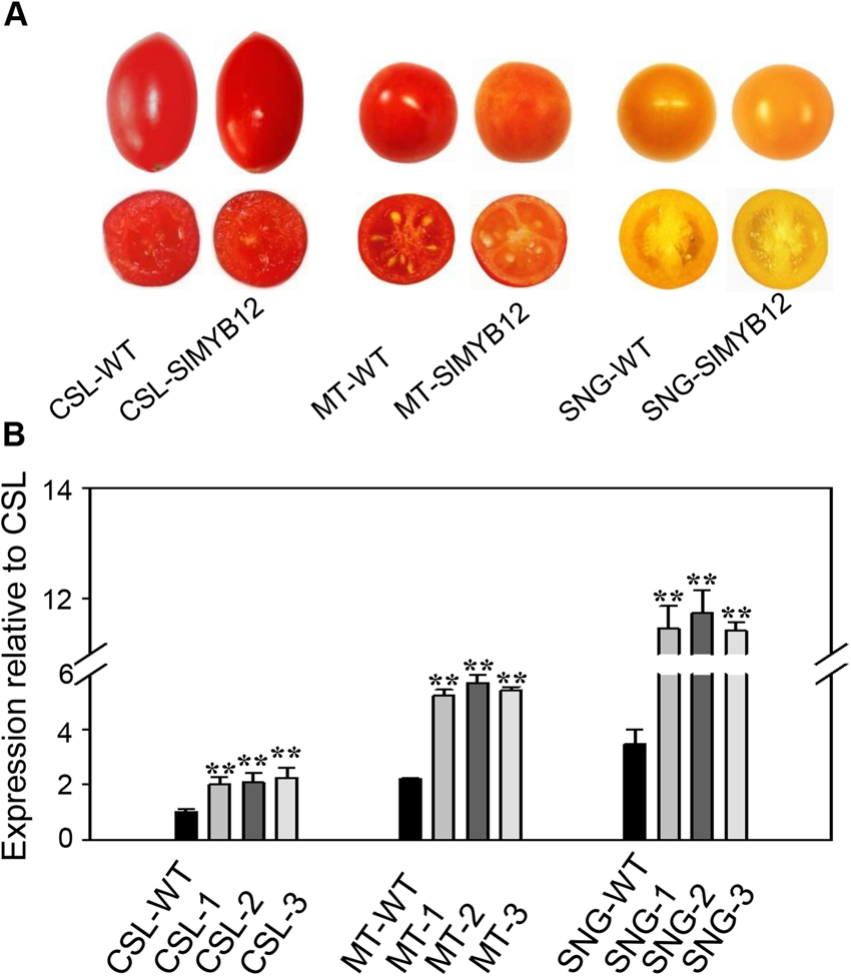
Phenotypes of wild-type and *SlMYB12*-overexpressing tomatoes. (**A**) Phenotype of *SlMYB12* expression in three tomato varieties. Control fruit (left) and *SlMYB12*-overexpressing plant (right). CSL-WT, Csl09-03 wild-type; CSL-SlMYB12, Csl09-03 *SlMYB12*-overexpressing tomato; MT, Micro-Tom wild-type; MT-SlMYB12, Micro-Tom *SlMYB12*-overexpressing tomato; SNG, Sheng Nv-Guo wild-type; SNG-SlMYB12, Sheng Nv-Guo *SlMYB12*-overexpressing tomato. (**B**) Relative expression levels of *SlMYB12* in control and T_1_ generation *SlMYB12* transgenic tomatoes. Each value was normalized to *SlMYB12* expression in wild-type Csl09-03 and is indicated as the mean ± standard deviation of three experimental replicates. CSL-1, CSL-2, CSL-3, MT-1, MT-2, MT-3, SNG-1, SNG-2, and SNG-3 indicate the T_1_ heterozygous lines of each variety. **P < 0.01.

To gain insight into the function of *SlMYB12* in flavonol biosynthesis in tomato, *Agrobacterium* strains containing the pX6-E8::*SlMYB12* (the digested full-length *SlMYB12* cDNA with the E8 tomato fruit-specific promoter in pX6 carrier) plasmids were transformed into three tomato varieties. Three T_1_ heterozygous lines of each variety were chosen for further study: CSL-1, CSL-2, CSL-3; MT-1, MT-2, MT-3; SNG-1, SNG-2, SNG-3. The expression levels of *SlMYB12* in each line were confirmed by qRT-PCR (Fig. 4B). By comparing with Csl09-03, the expression of *SlMYB12* in transgenic tomato lines CSL-1, CSL-2 and CSL-3 were increased almost 2.1-fold on average. By contrast, the expression of *SlMYB12* in Micro-Tom (MT-1, MT-2 and MT-3) showed an average of 2.47-fold increase compared to Csl09-03. The transgenic Sheng Nv-Guo lines showed a 3.5-fold increase compared to the non-transgenic lines and approximately an 11.54-fold increase compared to Csl09-03.

There were no visible differences between the transgenic lines and wild-type lines during any growth stage in Csl09-03 (Fig. 4A). Interestingly, visible differences between the *SlMYB12* overexpression lines and wild-type Micro-Tom and Sheng Nv-Guo were observed during the maturation stage. The fruit of Micro-Tom turned to red, while the transgenic line turned to red-orange, and the fruit of Sheng Nv-Guo normally showed orange skin, while the transgenic tomatoes turned to a visibly different orange-yellow (Fig. 4A).

**Figure 5:**
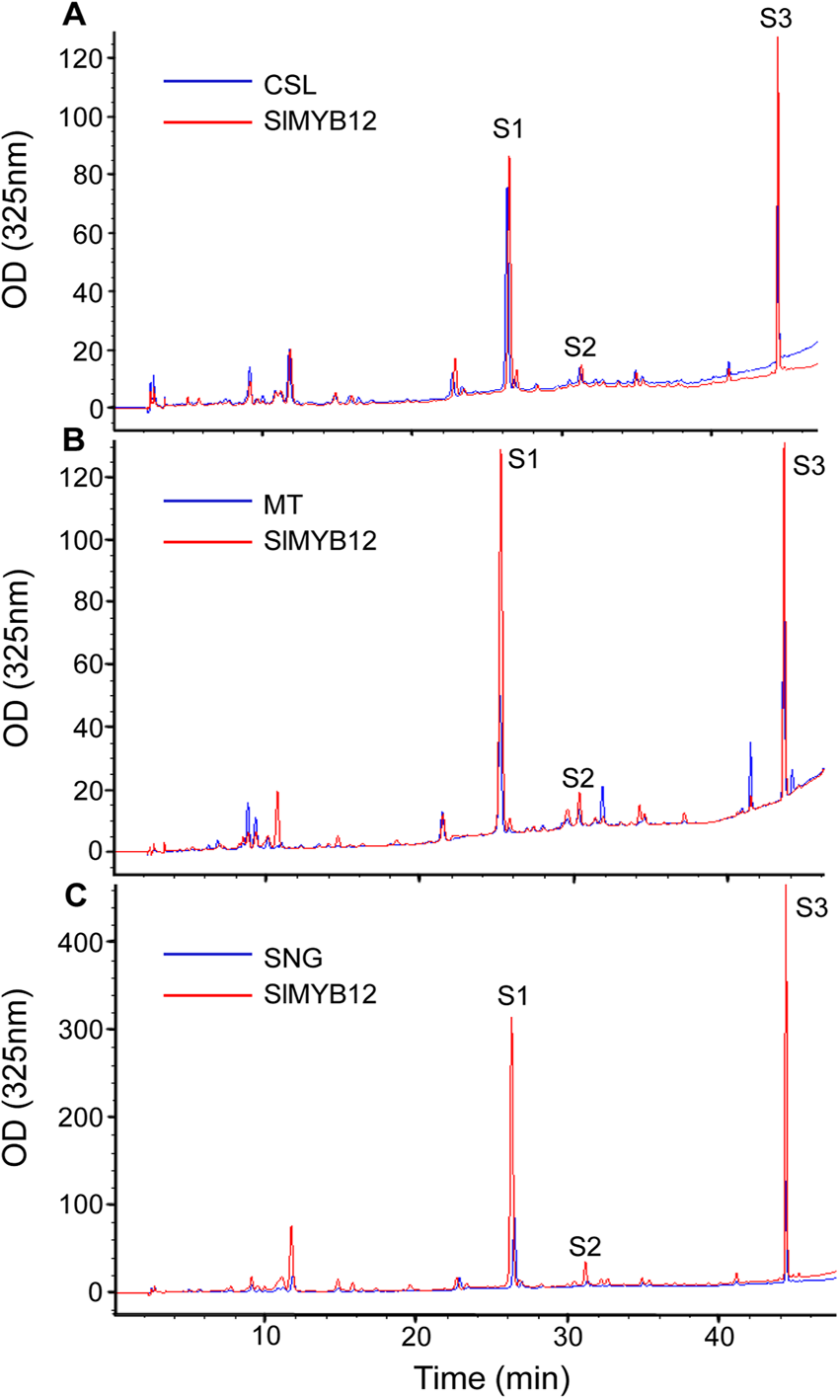
HPLC analysis of extracts of wild-type and *SlMYB12*-overexpressing tomatoes. (**A**) HPLC analysis of extracts from Csl09-03 wild-type (CSL) and Csl09-03 *SlMYB12*-overexpressing tomato (SlMYB12). (**B**) HPLC analysis of extracts from Micro-Tom wild-type (MT) and Micro-Tom *SlMYB12*-overexpressing tomato (SlMYB12). (**C**) HPLC analysis of extracts from Sheng Nv-Guo wild-type (SNG) and Sheng Nv-Guo *SlMYB12*-overexpressing tomato (SlMYB12). S1, quercetin rutinoside (rutin); S2, kaempferol rutinoside; S3, naringenin chalcone. Fruit skins of the three transgenic lines of each variety were mixed together for detection.

To confirm that the visible difference in *SlMYB12* overexpression lines was associated with flavonol content, HPLC analysis was performed to detect the contents of each individual flavonol in all lines. Generally, the concentrations of individual flavonols were increased to varying degrees in *SlMYB12*-overexpressing transgenic tomato fruits compared with those from wild-type lines (Table 2, Fig. 5). Overexpression of *SlMYB12* in variety Csl09-03 led to a 10.09- to 12.03-fold increase in rutin, a 2.0- to 3.21-fold increase in kaempferolrutinoside, and a 2.66- to 3.75-fold increase in naringenin chalcone compared to wild-type. In total, *SlMYB12* overexpression in Csl09-03 led to an average 5.07-fold increase in total flavonol content (2113.37 µg/g DW to 416.66 µg/g DW in wild-type) (Table 2, Fig. 5A). In the variety Micro-Tom, overexpression of *SlMYB12* resulted in a 16.25-fold increase in rutin, and the contents of kaempferolrutinoside and naringenin chalcone increased by 6.19- and 10.33-fold on average, respectively. In *SlMYB12*-overexpressing Micro-Tom, the average flavonol content (12481.38 µg/g DW) was approximately 11.18-fold higher than that in wild-type Micro-Tom (1116.12 µg/g DW) (Table 2, Fig. 5B). In *SlMYB12*-overexpressing Sheng Nv-Guo, the contents of total and individual flavonols were significantly increased (35644.82 µg/g DW total; 19.83-fold for rutin; 6.83-fold for kaempferolrutinoside; 3.92-fold for naringenin chalcone) compared with wild-type Sheng Nv-Guo (3368.48 µg/g DW) (Table 2, Fig. 5C). These results demonstrated that flavonol content was strongly associated with variation in the expression level of *SlMYB12* (correlation analysis R^2^ = 0.980).

**Table 2:** Quantification of major flavonols in three wild-type and *SlMYB12*-expressing cherry tomato variety peels.

	QueRut^g^ (μg g^−1^ DW^j^)	Fold increase	KaeRut^h^ (μg g^−1^ DW)	Fold increase	NC^i^ (μg g^−1^ DW)	Fold increase
CSL^a^	110.37 ± 0.25		30.15 ± 0.01		276.14 ± 5.89	
CSL-1^b^	1113.25 ± 10.47	10.09	60.42 ± 0.10	2.00	735.78 ± 10.56	2.66
CSL-2	1121.17 ± 2.30	10.16	96.78 ± 2.00	3.21	1035.88 ± 9.89	3.75
CSL-3	1327.58 ± 2.36	12.03	81.45 ± 0.02	2.70	767.80 ± 7.94	2.78
MT^c^	450.46 ± 0.17		230.40 ± 0.10		435.26 ± 3.23	
MT-1^d^	5581.45 ± 19.25	12.39	1330.75 ± 12.25	5.78	3943.54 ± 6.98	9.06
MT-2	7321.42 ± 15.66	16.25	1385.24 ± 11.52	6.01	4329.85 ± 7.77	9.95
MT-3	6780.48 ± 9.35	15.05	1560.19 ± 27.15	6.77	5211.22 ± 15.32	11.97
SNG^e^	1350.20 ± 1.50		330.69 ± 0.91		1687.59 ± 10.23	
SNG-1^f^	25991.35 ± 58.62	19.25	2175.94 ± 17.21	6.58	6098.60 ± 11.36	3.61
SNG-2	24590.47 ± 125.24	18.21	1930.86 ± 25.14	5.84	6291.41 ± 15.89	3.73
SNG-3	29732.15 ± 42.15	22.02	2670.27 ± 19.42	8.07	7453.43 ± 16.78	4.42

^a^CSL, wild-type (Csl09-03) tomato peels; ^b^CSL-1, 2,3, three lines of *SlMYB12*-expressing tomato (CSL variety); ^c^MT, wild-type (Micro Tom) tomato peels; ^d^MT-1, 2,3, three lines of *SlMYB12*-expressing tomato (MT variety); ^e^SNG, wild-type (Sheng Nv-Guo) tomato peels; ^f^SNG-1, 2,3, three lines of *SlMYB12*-expressing tomato (SNG variety); ^g^QueRut, quercetin rutinoside (rutin); ^h^KaeRut, kaempferolrutinoside; ^i^NC, naringenin chalcone; ^j^DW, dry weight.

### Overexpression of *SlMYB12* alters the expression of flavonol biosynthesis genes

**Figure 6:**
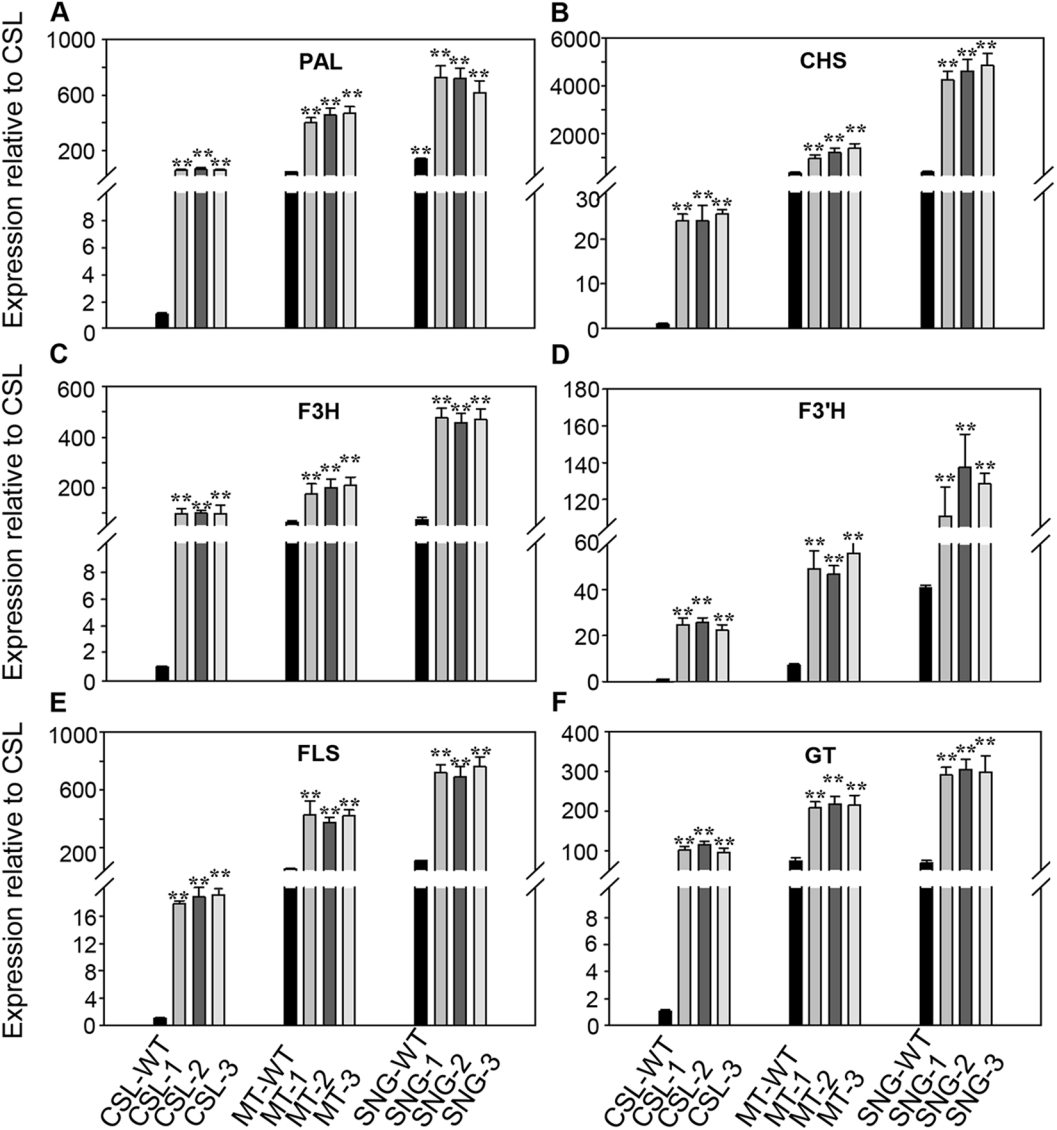
Analysis of transcript levels of the main genes in flavonol metabolism in wild-type and *SlMYB12*-overexpressing tomatoes by qRT-PCR. Analyzed genes are described in Fig. 1. (**A**) PAL, phenylalanine ammonialyase; (**B**) CHS, chalcone synthase; (**C**) F3H, flavanone-3-hydroxylase; (**D**) F3′H, flavonoid-3′-hydroxylase; (**E**) FLS, flavonol synthase; (**F**) GT, flavonol-3-glucosyltransferase. Two or three fruits were sampled from each plant. Each value was normalized to the gene expression in cultivar CSL (wild-type) and is indicated as the mean ± standard deviation of three experimental replicates. CSL-WT, Csl03-09 wild-type; MT-WT, Micro-Tom wild-type; SNG-WT, Sheng Nv-Guo wild-type. CSL-1, CSL-2, CSL-3, MT-1, MT-2, MT-3, SNG-1, SNG-2, and SNG-3 indicate the T_1_ heterozygous lines of each variety. **P < 0.01.

To confirm the correlation between the overexpression of *SlMYB12* and flavonol biosynthesis genes, quantitative real-time PCR (qRT-PCR) was performed in all obtained lines. In *SlMYB12*-overexpressing Csl09-03 tomatoes, 18.37- to 104.27-fold increases of flavonol biosynthesis genes were observed: PAL (62.30-fold), CHS (24.08-fold), F3H (97.74-fold), F3′H (24.31-fold), FLS (18.34-fold) and GT (104.27-fold) (Fig. 6). Comparing with Csl09-03, in *SlMYB12*-overexpressing Micro-Tom fruits (Fig. 6): PAL was up-regulated 441.69-fold, CHS 1171.28-fold, F3H 194.16-fold, F3′H 50.52-fold, CSL 410.62-fold, and GT 213.70-fold. In *SlMYB12*-overexpressing transgenic Sheng Nv-Guo tomatoes: PAL was increased 688.12-fold, CHS was increased 4702.38-fold, F3H, F3′H, FLS and GT were increased 468.52-, 125.61-, 723.93- and 298.15-fold, respectively (Fig. 6). These results showed that the overexpression of *SlMYB12* altered the expression of flavonol biosynthetic genes in all three varieties, particularly inducing the highest expression of these genes in Sheng Nv-Guo fruits rather than in Micro-Tom and Csl09-03.

Taking the wild-type tomato varieties as controls, PAL was up-regulated 62.30-fold in Csl09-03 transgenic tomato (Csl-T), 9.93-fold in Micro-Tom transgenic tomato (MT-T), and 5.02-fold in Sheng Nv-Guo transgenic tomato (SNG-T) (Fig. 6A); CHS showed a 24.08-fold up-regulation in Csl-T, 3.53- and 12.23-fold in MT-T and SNG-T (Fig. 6B). F3H and F3′H were increased 97.74- and 24.31-fold in Csl-T, 3.10- and 7.02-fold in MT-T, and 6.44- and 3.08-fold in SNG-T, respectively (Fig. 6C,D). Furthermore, we found that the expression of FLS increased 18.34-fold in Csl-T, 7.91-fold in MT-T, and 6.55-fold in SNG-T (Fig. 6E). The expression of GT was up-regulated approximately 104.27-fold in Csl-T, 3.13- in MT-T and 3.80-fold in SNG-T (Fig. 6F). These results indicated that the overexpression of *SlMYB12* increases the expression of biosynthetic genes, which may lead to the increased flavonol contents in different varieties.

### Overexpression of *SlMYB12* increased antioxidant capacity

**Figure 7:**
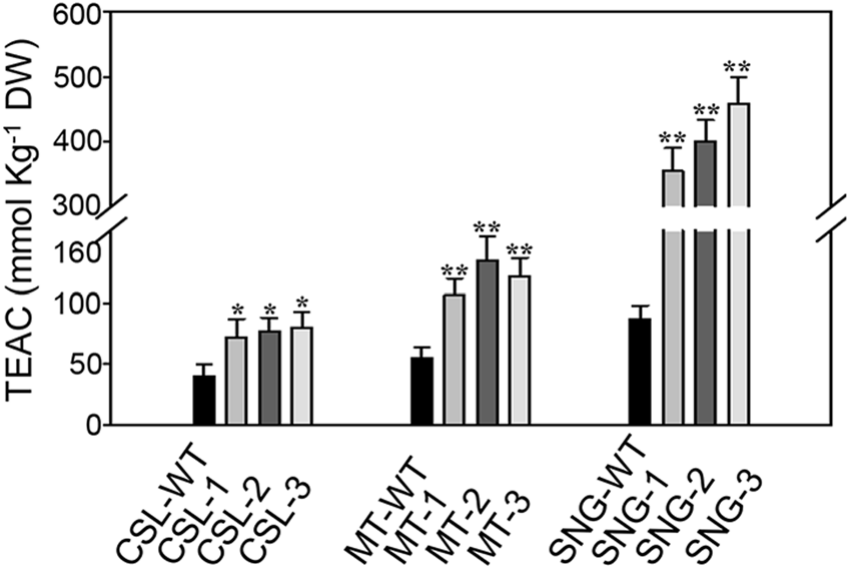
Total antioxidant capacity in wild-type and *SlMYB12*-overexpressing tomatoes. Fresh and skin antioxidant activities in mature tomato fruits of each cultivar. Three different tomato fruits of each cultivar were pooled for detection. CSL-WT, Csl03-09 wild-type; MT-WT, Micro-Tom wild-type; SNG-WT, Sheng Nv-Guo wild-type. CSL-1, CSL-2, CSL-3, MT-1, MT-2, MT-3, SNG-1, SNG-2, and SNG-3 indicate the T_1_ heterozygous lines of each variety. Each value represents repeated four times independent experiments, and the vertical bars expressed the arithmetic means ± standard deviations (SD). **P < 0.01, *P < 0.05.

The antioxidant capacity of three varieties and transgenic lines were measured by the TEAC method. In comparison to the variety Csl09-03, overexpression of *SlMYB12* in Csl09-03 showed a 1.91-fold increase, overexpression of *SlMYB12* in Micro-Tom showed a 3.03-fold increase, and approximately a 10.12-fold increase was observed in the Sheng Nv-Guo transgenic line (Fig. 7). Moreover, overexpression of *SlMYB12* in Csl09-03 led to a 1.91-fold increased antioxidant capacity compared to its wild-type. Similarly, 2.21-fold and 4.65-fold increases were found in *SlMYB12* transgenic lines in Micro-Tom and Sheng Nv-Guo compared with their wild-type cultivars (Fig. 7). Overall, overexpression of *SlMYB12* can increase antioxidant capacity, which may be associated with the flavonol contents in three tomato varieties (correlation analysis R^2^ = 0.974).

## Discussion

Flavonols are considered important phytochemicals in cherry tomato due to their high antioxidant and cell-modulating properties, which support human health^38–40^. However, the mechanism by which flavonol content is regulated in different tomato varieties is still unclear. In a previous study, expression of *AtMYB12* was shown to increase flavonol content in Micro-Tom and Money Maker^7^. This study investigated the role of *SlMYB12* in flavonol biosynthesis in three tomato varieties. In these three varieties, Sheng Nv-Guo showed the highest expression of *SlMYB12* and the highest flavonol content, while Csl09-03 contained the lowest flavonol content and had the lowest expression of *SlMYB12* (Table 1, Fig. 1). Based on the correlation coefficient R^2^ = 0.922 (expression level of *SlMYB12* and flavonol content) in Csl09-03, Micro-Tom and Sheng Nv-Guo, as well as the correlation coefficient R^2^ = 0.929 in ten additional tomato cultivars, we propose that *SlMYB12* is a good potential marker gene for flavonol content assessment among tomato varieties. Overall, *SlMYB12* provides a theoretical and practical basis for tomato variety selection based on flavonol content.

*SlMYB12* showed diverse expression levels after overexpression in three tomato cultivars, which also led to increased concentrations of individual flavonols compared to each wild-type plant variety (R^2^ = 0.980). Rutin increased 10.76-fold in Csl09-03, 14.56-fold in Micro-Tom, and 19.83-fold in Sheng Nv-Guo. Similarly, a 2.64-fold increase in kaempferolrutinoside was found in Csl09-03, while 6.19-fold and 6.83-fold increases were found in Micro-Tom and Sheng Nv-Guo (Table 2). When *SlMYB12* was overexpressed, the content of naringenin chalcone was increased 3.06-fold in Csl09-03 and 10.33-fold in Micro-Tom, and the amount of the increase coincided with the amount of increase in *SlMYB12* expression. The visible phenotypic differences in color may be due to the higher expression level of *SlMYB12* and the consequent increase in flavonol concentrations, especially rutin and naringenin chalcone, which have been reported to regulate the yellow color of fruit^7,33^.

The color of tomato fruit is interrelated with many substance contents, such as lycopene, carotenoids, flavonols, anthocyanin, and so on. With the prerequisite of wild-type tomato variety, the content of lycopene, carotenoid substances and their derivatives, are the main pigments that determine fruit coloration in tomato fruit. According to the results of previous studies, the fruit with orange color contains less content of lycopene but high content of carotenoid, the fruit with pink color contains a small amount of lycopene, and only minute quantity of carotenoids, the fruit with bright yellow color contains a small amount of carotenoids and a fat lot of lycopene^41^. In our results, on the basis of single variable, the fruit of Micro-Tom transgenic line turned to red-orange (12481.38 µg/g DW total, 11.18-fold higher), the fruit of Sheng Nv-Guo transgenic tomatoes turned to a visibly different orange-yellow (35644.82 µg/g DW total, 10.58-fold higher), and the fruit of Csl09-03 transgenic tomatoes (2113.37 µg/g DW, 5.07-fold higher) showed the same coloration as the wild-type. The fruit coloration was regulated by the high level content of flavonols, such as in Micro-Tom and Sheng Nv-Guo transgenic tomatoes, but not in the less increased level in Csl09-03 transgenic tomatoes. For example, *SlMYB12*-silenced tomatoes created by VIGS (virus-induced gene silencing) led to a pink-colored tomato phenotype^33^. The fruit turns orange instead of red in the *AtMYB12* expressing tomato fruit, is because of the increasing content of rutin^7^. On the basis of a tomato variety, *SlMYB12* is a R2R3-MYB transcription factor^41^, which may regulate the contents of flavonols, such as naringenin chalcone, as a flavonol-specific activator associated with the color of tomato skin.

Through Blast analysis, *SlMYB12* was shown to have 80% identity in amino acid sequence with *AtMYB12*^7^. Our study demonstrates that *SlMYB12* acts as a positive regulator, similar to *AtMYB12*, in the flavonol biosynthetic pathway. Luo *et al*.^7^ demonstrated that *AtMYB12* increased rutin by 33.6-fold, kaempferolrutinoside by 209-fold, and naringenin chalcone by one fold in transgenic Micro-Tom tomato fruit. However, *SlMYB12* overexpression led to a greater increase in naringenin chalcone (10.33-fold) and smaller increases in rutin (14.56-fold) and kaempferolrutinoside (6.19-fold) in Micro-Tom (Table 2). Naringenin chalcone is mainly synthesized from 4-coumaroyl-CoA and 3*malonyl CoA in a reaction catalyzed by chalcone synthase (CHS) (Fig. 1). qRT-PCR results indicated that the transcript levels of *CHS* were the highest among flavonol biosynthesis genes in *SlMYB12*-overexpressing tomato (Fig. 6). However, PAL was found to be the highest expressed gene in *AtMYB12*-expressing tomato^14^. This difference in the increased level of naringenin chalcone may be due to differences in the increases in CHS transcript levels in *SlMYB12* and *AtMYB12* transgenic plants. *CHS* may be mainly regulated by *SlMYB12*, rather than by other flavonol biosynthetic genes, and only one among a group of genes regulated by *AtMYB12*^7,33^. Based on these findings, we considered that the overexpression of *SlMYB12* increased the levels of the intermediate compound of flavonol biosynthesis pathway, naringenin chalcone, and this increase depended on the induction of *CHS*. The other flavonol biosynthetic genes (Fig. 1) were induced as well, but not at such a high level as *CHS*, indicating that *CHS* was one of the target genes of *SlMYB12* and was important for the efficacy of *SlMYB12* in tomato (Figs 5 and 6). In addition to CHI and PAL (the first gene in the general phenylpropanoid pathway), F3′H was induced in transgenic tomato. A mutation in the *Arabidopsis F3′H* gene results in altered localization of *CHS*, indicating that *F3′H* may be a part of a membrane anchor for *CHS* in the flavonol pathway^42,43^^.^ Moreover, F3′H also controls the major pathway to compose kaempferolrutinoside from dihydro kaempferol (Fig. 1). We suggest that F3′H may be another major enzyme regulated by *SlMYB12* in tomato.

In conclusion, we demonstrate here the effects of *SlMYB12* expression in three different wild-type tomato varieties and show its positive function on the flavonol biosynthesis pathway through comparison of flavonol contents, biosynthetic pathway gene expression levels, and antioxidant capacities in *SlMYB12-*overexpressing tomato plants. The endogenous gene *SlMYB12* promoted by the tomato fruit-ripening-specific promoter E8 produces a functional vegetable with higher nutritional value, with similar production and phenotype, wider public acceptance, and lower risk assessment. Moreover, we suggest *SlMYB12* expression level as an index of flavonol content for tomato variety selection in genetic improvement methods.

## Methods

### Plant materials and growth conditions

Seeds of three cherry tomato variety inbred lines, Micro-Tom, CSl09-03 and Sheng Nv-Guo, were grown in a glass greenhouse under a 16 h light/8 h dark cycle at 25 °C, with 70% relative humidity.

We obtained fruits of ten additional wild-type cherry tomato cultivars (numbered from 1 to 10) from Yantai academy of agricultural sciences. Cultivar 1: CC-4, Cultivar 2: GX-1, Cultivar 3: KDL-a, Cultivar 4: KD-198, Cultivar 5: FBB-c, Cultivar 6: XG-4, Cultivar 7: OG-d, Cultivar 8: YF-e, Cultivar 9: P15, Cultivar 10, M-82.

The full-length cDNA of *SlMYB12* (EU419748) was amplified from *Solanum lycopersicum* Micro-Tom with *SlMYB12* F1 (5′-ATGACTAGTATGGGAAGAACACCTTGTTG-3′) and R1 (5′-ATGACTAGTCTAAGACAAAAGCCAAGATACAA-3′) by RT-PCR (the *Spe*I site is underlined), then digested with *Spe*I. The E8 promoter was amplified from *Solanum lycopersicum ver*. Zhongshu No.4 using the primer pair E8PF1 (5′-ATGCTCGAGAGGAATTTCACGAAATCG-3′; the *XhoI* site is underlined) and E8PR1 (5′-ATGACTAGTTCTTTTGCACTGTGAATG-3′; the *Spe*I site is underlined) according to the sequence deposited in GenBank (13437). The DNA of the E8 promoter was digested with *Xho*I and *Spe*I and then ligated into *Xho*I/*Spe*I digested pX6, replacing GFP, to produce the transitional vector pX6-E8. The digested full-length *SlMYB12* cDNA was inserted into pX6-E8 to produce the transitional vector pX6-E8::*SlMYB12*. This construct was transformed into *A. tumefaciens* strain AGL1 by electroporation. *Agrobacterium*-mediated transformation into tomato cotyledon explants (*S. lycopersicum* cv. Micro-Tom, CSl09-03 and Sheng Nv-Guo) was performed using a previously published method^31,44^. The presence of full-length *SlMYB12* DNA in *SlMYB12* transgenic tomato was confirmed by PCR with the E8PF1 (5′-ATGCTCGAGAGGAATTTCACGAAATCG-3′) and *SlMYB12* R1 (5′-ATGACTAGTCTAAGACAAAAGCCAAGATACAA-3′) primer pair. Ten positive independent plants was obtained in T_0_ generation for each tomato variety, and three transgenic lines in each variety (CSL-1, CSL-2, CSL-3; MT-1, MT-2, MT-3; SNG-1, SNG-2, SNG-3) in the T_2_ generation were used for further analysis.

### Quantification of flavonoids

The major flavonols in tomato fruits of Micro-Tom, CSl09-03 and Sheng Nv-Guo were extracted from freeze-dried tomato test specimens using 70% methanol from Sigma (dry-weight basis), and the major flavonols from the ten tomato cultivars numbered 1 to ten were extracted from skin samples (0.2 mg) using 2 ml of 100% methanol from Sigma (http://www.sigmaaldrich.com/) (fresh-weight basis). The flavonols were quantified by HPLC (high-performance liquid chromatography, Agilent Technologies 1200 series) with acolumn (Agilent Technologies ZORBAX SB-C18 4.6*250 mm). A gradient elution was performed with solvent A consisting of 3% acetonitrile and 10% formic acid and solvent B consisting of acetonitrile (50%) and formic acid (10%), with the following elution program: 0 min 4% B, 20 min 20% B, 35 min 40% B, 40 min 60% B, 45 min 90% B, 55 min 4% B, flow rate of 1 mL/min^7^. Detection by ultraviolet (UV) chromatograms was recorded at 325 nm. All flavonol standards, rutin, kaempferolrutinoside and naringenin chalcone were obtained from either Sigma-Aldrich or Extrasynthèse (Genay, France).

### Quantitative real-time PCR (q RT-PCR)

The concentration and purity of the RNA samples were determined by UV absorbance spectrophotometry (260 nm/280 nm ratio). First-strand cDNA was synthesized using Super Quick RT MasterMix (CWBio, China) following the manufacturer’s instructions. Transcription of phenylpropanoid biosynthetic genes wasanalyzed by quantitative PCR using gene-specific primers (Table S1)^45^. All target gene confirmations were performed using SYBR Premix Ex Taq (TaKaRa, Dalian, China). All tomato quantifications were normalized to the abscisic stress ripening gene 1 (*ASR1*, accession no. L08255.1); these genes were amplified under the same conditions. Quantitative PCR was conducted on the Bio-Rad iQTM5 Light Cycler analysis system with SYBR^®^ Premix Ex TaqTM (Tli RNase H Plus). The PCR program was as follows: 95 °C for 30 s, followed by 40 cycles of 95 °C for 5 s, 55 °C for 20 s, and 72 °C for 30 s. A heat dissociation curve (55–95 °C) following the final cycle of the PCR was performed to test the specificity of the PCR amplification. The relative quantification analysis was performed by relative standard curve according to the threshold values (Ct) generated. The *ASR1* gene was used as an internal control to standardize the results. We mixed plant tissues from all three T_1_ progeny together to detect the expression of phenylpropanoid biosynthetic genes between the varieties of tomato and the different transgenic tomato lines. All experiments were carried out with three biological repeats and four technical trials.

### Total antioxidant activity

Freeze-dried tomato fruit samples (50 mg) were extracted with 70% ethanol, and the antioxidant capacity of the extracts was analyzed. To measure antioxidant capacity, we performed the 2,2′-azinobis (3-ethylbenzothiazoline-6-sulfonic acid) (ABTS)/Trolox equivalent antioxidant capacity (TEAC) assay, which measures the ability of compounds to scavenge the ABTS radical cation (ABTS^+^) in relation to Trolox (6-hydroxy-2,3,7,8-tetramethylchroman-2-carboxylic acid; Sigma). The results were expressed as the TEAC in mmol of Trolox per kg of dry weight^46,47^. All experiments were carried out with three biological repeats and three technical trials.

### Statistical Analyses

Each value represents repeated independent experiments, and the vertical bars expressed the arithmetic means ± standard deviations (SD). Tukey’s test was used to calculate statistical significance, and the significant differences between treatments and the untreated control are represented by * at P < 0.05 and ** at P < 0.01.

## Electronic supplementary material

Dataset 1
