# Supplementary material for: SlMYB12 Regulates Flavonol Synthesis in Three Different Cherry Tomato Varieties
Source: Sci Rep. 2018 Jan 25;8:1582. doi: 10.1038/s41598-018-19214-3 (PMC5785513; doi:10.1038/s41598-018-19214-3)

***SlMYB12* Regulates Flavonol Synthesis in Three Different Cherry Tomato Varieties**

Shaoli Wang^1,4^ , Zhaohui Chu^1,4^, Ru Jia^1^, Dan Fei^2^, Xiangling Shen^3^, Yang Li^1*^&Xinhua Ding^1*^

^1^ State Key Laboratory of Crop Biology, Shandong Provincial Key Laboratory for Biology of Vegetable Diseases and Insect Pests, Shandong Agricultural University, Taian 271018, Shandong, P. R. China

^2^Anhui Biothun Biotechnology Company, Hefei, Anhui, 230088, PR China.

^3^Biotechnology Research Center, China Three Gorges University, Yichang City, Hubei, 443002, PR China.

^4^These authors contributed equally to this work.

*Correspondence:

Yang Li

Tel: 86-538-8245569

Fax: 86-538-8249913

Email:younuowanwan@126.com

Xinhua Ding

Tel: 86-538-8245569

Fax: 86-538-8249913

Email:xhding@sdau.edu.cn

**Table S1.** Primers used for real-time RT-PCR of genes in flavonol biosynthetic pathway in tomat**o.**

| Primer | Gene | Sequence (5' to 3') |
| --- | --- | --- |
| SlMYB12_F | *SlMYB12* | GAGCAATAATGTAGGGAATAG |
| SlMYB12_R | *SlMYB12* | TTGAAGTAAGTTAGTGTCAGTAT |
| PAL_CSL_F | *PAL* | AACCTATCTCGTGGCTCTTT |
| PAL_CSL_R | *PAL* | TCTTTTTCGCTGAATCTTGC |
| C4H_CSL_F | *C4H* | CAACAGAAAGGAGAGATCAACGAG |
| C4H_CSL_R | *C4H* | CACAGCCTGAAGGTATGGAAGC |
| 4CL_CSL_F | *4CL* | ACACACAAAGGCTTAGTCACGA |
| 4CL_CSL_R | *4CL* | AACAGAGGCAACACACACATCA |
| CHS_CSL_F | *CHS* | TGGTCACCGTGGAGGAGTATC |
| CHS_CSL_R | *CHS* | GATCGTAGCTGGACCCTCTGC |
| CHI_CSL_F | *CHI* | GTTTTTCACAAACCAACAGTTCTGAT |
| CHI_CSL_R | *CHI* | GAAGCAGTGCTCGATTCCATAAT |
| F3H_CSL_F | *F3H* | CACACCGATCCAGGAACCAT |
| F3H_CSL_R | *F3H* | GCCCACCAACTTGGTCTTGTA |
| F3'H_CSL_F | *F3'H* | GCACCACGAATGCACTTGC |
| F3'H_CSL_R | *F3'H* | CGTTAGTACCGTCGGCGAAT |
| FLS_CSL_F | *FLS* | GAGCATGAAGTTGGGCCAAT |
| FLS_CSL_R | *FLS* | TGGTGGGTTGGCCTCATTAA |
| GT_CSL_F | *GT* | CGAACGACGAAACACTGTTGA |
| GT_CSL_R | *GT* | TGCAGCATAGATGGCATTGG |
| ASR1_CSL_F | *ASR1* | CCTGTTCCACCACAAGGACAA |
| ASR1_CSL_R | *ASR1* | GTGCCAAGTTTACCGATTTGC |

**Table S2.** The expression of *SlMYB12* and the content of flavonol in ten wild-type cherry tomato variety peels

| Cultivar^a^ | Expression level^b^ | Flavonol content  (µg g^-1^ FW^c^) |
| --- | --- | --- |
| 1 | 1±0.15 | 469.64±8.96 |
| 2 | 2.64±0.38 | 617.04±12.87 |
| 3 | 25.96±4.62 | 4688.79±25.84 |
| 4 | 4.12±0.69 | 1876.54±11.75 |
| 5 | 4.82±0.57 | 1920.38±6.87 |
| 6 | 14.89±1.7 | 5887.66±30.26 |
| 7 | 50.74±6.3 | 10210.16±64.82 |
| 8 | 18.61±2.4 | 4756.82±10.59 |
| 9 | 12.37±2.09 | 3030.31±5.86 |
| 10 | 56.62±5.21 | 15037.83±53.85 |

^a^Cultivar, ten different wild-type tomato cultivars were numbered from 1 to 10; ^b^Expression level, the expression of *SlMYB12* in the variety 2 to 10 were compared with the variety 1; ^c^FW, fresh weight.

**Figure S1. Correlation analysis of *SlMYB12* expression level and flavonol contents in ten wild-type tomato varieties.** Each value of *SlMYB12* expression level was normalized to the gene expression in cultivar 1 and is indicated as the mean ± standard deviation of three experimental replicates. Fruit skins of each cultivar were mixed together for detection based on fresh weight.


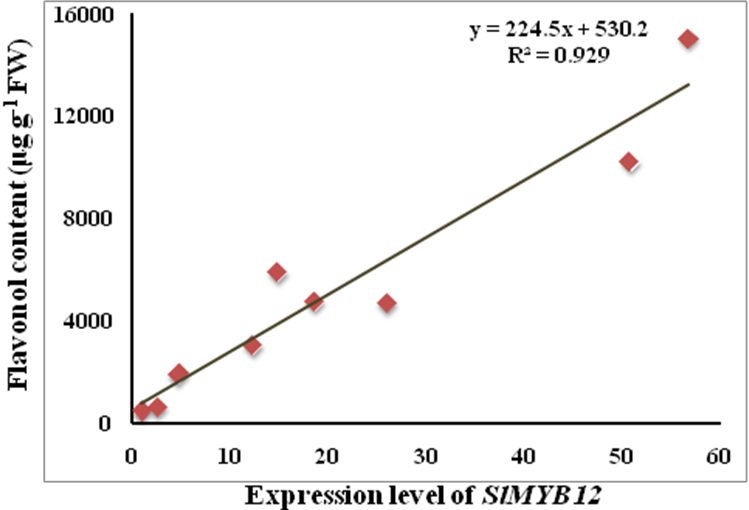

Supplement: Supplementary file 1 — Dataset 1 [file 41598_2018_19214_MOESM1_ESM.doc]
